# Will the traditional horticultural breeding and genetics research be fairly valued in academia?

**DOI:** 10.1038/hortres.2015.53

**Published:** 2015-10-28

**Authors:** Zong-Ming (Max) Cheng, Dennis J Werner

**Affiliations:** 1 Editor-in-Chief, *Horticulture Research*; 2 The Laboratory of Fruit Crop Systems Biology, College of Horticulture, Nanjing Agricultural University, Nanjing, Jiangsu Province 210095, The People's Republic of China; 3 Department of Plant Sciences, University of Tennessee, Knoxville, TN 37996, USA; 4 Department of Horticultural Science, North Carolina State University, Box 7609, Raleigh, NC 27695-7609, USA

I wrote the inaugural Editorial when *Horticulture Research* was launched in January 2014. This second Editorial was trigged by the manuscript that was submitted to *Horticulture Research* (HORTRES.2015.49, www.nature.com/articles/hortres201549), a summary of 17 years of traditional genetics on a woody ornamental tree called redbud.

I have always been fascinated by the wealth of unique ornamental trees, mostly caused by genetic mutations. Since the redbud and dogwood are the two signature ornamental trees in eastern Tennessee, where University of Tennessee locates, I called Dr Dennis Werner and asked him to write a story behind this research and how he could continue and sustain this research for 17 years.

In his story (see below), I see several important questions that are in need of open discussions. Over the past 25 years as a faculty in land-grant universities in the United States, I have seen eliminations or switching from the traditional breeding and genetics research programs to biotechnology, molecular biology and genomics, and alike. University administrators are shifting funds and changing evaluation matrixes, and have been placing more emphasis on publications, grants, and high impact factor journal articles. During the six years of my joint appointment at Nanjing Agricultural University, China, I have been struck by the “typhoon” of publishing high impact factor journal articles and changing evaluation matrix that swept through the Chinese research institutions. I have contacted many faculty members in the United States and China, and asked the following questions: “Will the traditional breeding and genetics (or similar programs) be fairly valued in academia, especially in land grant, or similar mission-oriented universities where horticultural research is primarily conducted?” “In the current academic environment, how can young scientists set long-term research goals without worrying about short-term pressures of publishing?” “How do we evaluate high impact research and does it equal to high impact factor journal articles?” As the Editor-in-Chief, I accepted Dr Werner’s article, which has taken 17 years to collect and aggregate the data, in *Horticulture Research*, to offer my view and support on this solid, traditional genetics research. I invite and encourage faculty and administrators to openly discuss these questions which may greatly impact research and service we perform to the horticultural science, horticultural industry, and ultimately our life in general.

## A 17-year story behind the new redbud trees

I began breeding efforts in *Cercis canadensis* (eastern redbud) in 1998, after 20 years of research in peach breeding. In many areas of the eastern United States, *C. canadensis* is a very popular landscape tree. In spite of its popularity, little breeding effort had been devoted to this species. Although myriad phenotypic variants exist in eastern redbud for leaf color, flower color, architecture, and plant size, I was surprised when examining the scientific literature that no reports of the genetics of these traits existed. At that time, I decided to initiate a long-term research effort aimed at not only developing new cultivars of redbud, but also accumulating data on the genetics of the major phenotypic variants in the species. Already a tenured Full Professor, I appreciated that I was in a position to embark on such a long-term project with a woody perennial species, perhaps a more difficult quest for a young plant breeding faculty member cognizant of fulfilling the expectations of grant and publication metrics. In this era of molecular genetics, I still enjoy engaging in the long-term process of applied plant breeding and cultivar development. Such long-term activities are more difficult to emphasize for young faculty today, as institutional priorities often are not compatible with this type of research. Unfortunately, institutional patience and long-term vision are often limited.


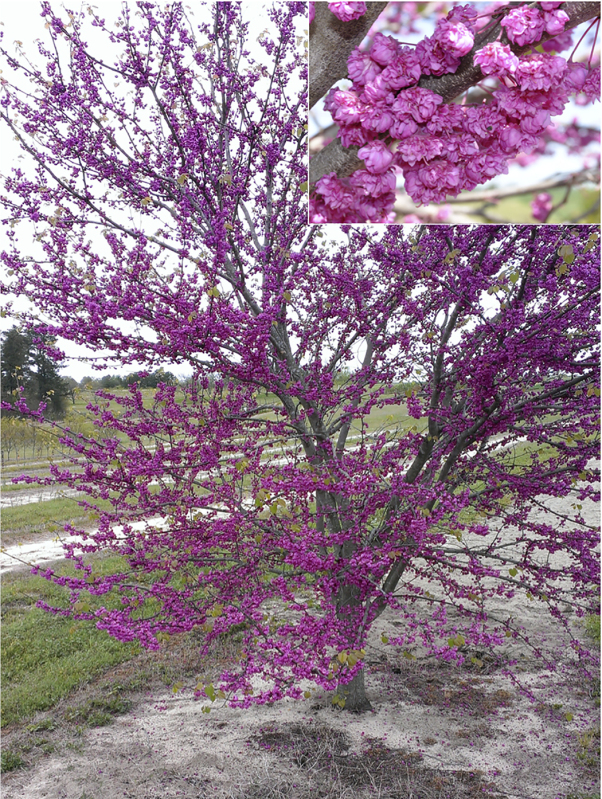


I have the good fortune of being a faculty member at North Carolina State University, where late Director J.C. Raulston of the North Carolina State Arboretum (now the JC Raulston Arboretum) accumulated what was then in the mid-1990s the most comprehensive collection of *Cercis* in the United States. His collection encompassed not only numerous forms and cultivars of *C. canadensis*, but all Asian and Eurasian species as well. Observing that variation, and appreciating the potential for creating new and improved landscape forms, inspired me to begin my redbud breeding efforts. Not only did I endeavor to develop new cultivars of redbud (‘Ruby Falls’, ‘Merlot’, ‘Whitewater’, and ‘Pink Pom Poms’ released to date), but early on my goal was also to contribute to the genetic knowledge of the species by making the appropriate hybridizations to elucidate the inheritance of the extant phenotypic variants. Interestingly, of the variants studied in this manuscript, three of the mutants did not even exist in 1998, but rather were discovered subsequent to the initiation of my studies.


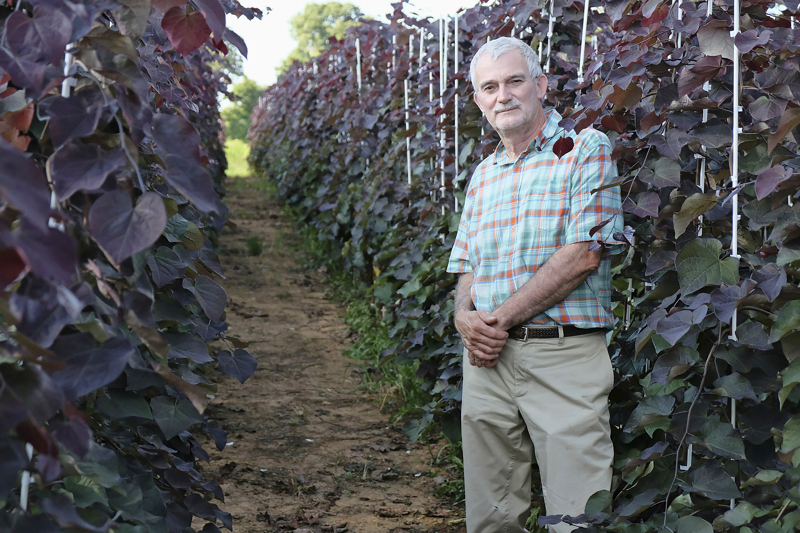


Hence, after 17 years of hybridizations, growing, and taking data on thousands of seedlings, and recently recruiting a graduate student who was interested in analyzing this wealth of data and executing the final controlled crosses to complete the effort, I thought it was time to finally share these results. Some may look upon these types of studies as dated, lacking in impact relative to more advanced molecular studies. However, I feel strongly that documentation of the existence and genetics of these mutants is critical, as many of them may prove to be excellent subjects for future advanced molecular and physiological studies. And of course, the data shared in this manuscript enhances our knowledge of *Cercis*, phylogenetically a basal member of the family Fabaceae.

